# Academic resilience and motivational beliefs in science among native and immigrant students in Türkiye: evidence from TIMSS 2019

**DOI:** 10.3389/fpsyg.2026.1838607

**Published:** 2026-07-29

**Authors:** Nurcan Kahraman

**Affiliations:** Department of Mathematics and Science Education, Bursa Uludag University, Bursa, Türkiye

**Keywords:** academic resilience, science education, self-concept, task value, TIMSS

## Abstract

Academic resilience is a critical factor in understanding the disparities in student achievement, particularly among disadvantaged groups. This study examined the level of academic resilience in science among native and immigrant students in Türkiye, using data from TIMSS 2019. In addition, the study investigated the relation of motivational beliefs (self-concept and task value) to science achievement of non-resilient students, based on Expectancy-Value Theory. Findings revealed that academic resilience is significantly lower among immigrant students compared to their native counterparts. Only 3.1% of low-SES immigrant students demonstrated resilience in science, while this proportion was 17% among low-SES native students. Fisher’s exact test suggested a statistically significant association between immigrant status and academic resilience (*p* = 0.003, OR = 0.11). Furthermore, the regression results indicate that self-concept was positively associated with science achievement for both groups, whereas task value was not significantly associated with achievement. However, the interaction analysis suggested a positive association for native students, while it revealed a negative association for immigrant students. This implies that high self-concept and high task value may be associated with additional challenges for immigrant students, possibly due to linguistic and cultural barriers.

## Introduction

Due to its potential for reducing disparities in academic achievement and promoting educational equity, academic resilience has garnered greater attention (Martin, 2002). Academic resilience is a concept that refers to the academic success of disadvantaged students despite the difficulties that they face. This concept relies on two main dimensions: risk and positive additivity. While risk is related to personal (e.g., ethnicity) or social (e.g., poverty) issues, positive additivity is related to unexpectedly positive outcomes (Tudor and Spray, 2017). Regarding academic resilience, generally, students’ academic performance is considered as the outcome (OECD, 2011). In other words, academic resilience means achieving more than expected despite limited socio-economic resources. These challenges may include socioeconomic hardships, familial issues, or systemic inequalities within educational frameworks (Waxman et al., 2003). Resilience, however, is not a fixed trait; rather, it is a dynamic process shaped by interactions between individuals and their environments (Ungar et al., 2007). Therefore, by understanding academic resilience, educators and policymakers can identify strategies to support at-risk students, thereby fostering inclusive learning environments.

Immigrant students illustrate a prominent example of risk and adaptation processes in terms of academic resilience. Migration is widely regarded as a complex risk factor since it is characterized by a combination of individual and environmental challenges. Immigrant students generally face language barriers, economic difficulties, cultural adaptation, and discrimination in educational systems (Suárez-Orozco et al., 2010). For instance, In the Turkish context, Syrian refugee students have been reported to face significant language barriers and social exclusion in public schools, with institutional factors such as school leadership and language support programs playing a critical role in their adaptation (Çelik and İçduygu, 2019). Relevant literature suggests that immigrant students generally have lower academic performance and show higher dropout rates (Heath et al., 2008). While immigrant students face unique challenges that can hinder their academic performance, similar disparities are also observed among disadvantaged groups within native student populations (OECD, 2011), highlighting the universal relevance of academic resilience in addressing educational inequities.

However, studies consistently found lower academic resilience rates among immigrant-origin students compared to their native peers; for instance, Gabrielli et al. (2022) demonstrated this pattern across seven European countries using PISA data, finding that school environment played a particularly decisive role in immigrant students’ outcomes. In this respect, academic resilience is becoming a key concept in the pursuit of equity in education. Therefore, this study aims to examine the differences in academic resilience between immigrant and native students in Türkiye, which can provide valuable insights for designing educational policies. While immigrant students often face a disadvantaged starting point in terms of academic resilience, native students may also be influenced by various motivational and environmental factors. In this regard, studying the impact of motivational factors, especially on non-resilient students, can pave the way for developing strategies to close educational achievement gaps. Understanding the factors influencing academic resilience requires a robust theoretical foundation. In this context, the Expectancy-Value Theory provides a valuable framework for exploring how students’ beliefs about their abilities and the value they place on academic tasks interact to effect their resilience and academic outcomes, especially under challenging circumstances. Indeed, Zhang and Bae (2020), in a systematic review of TIMSS studies, identified Expectancy-Value Theory as the motivational framework most closely aligned with the data available in the TIMSS student questionnaire.

### Theoretical framework

Expectancy-value theory presents a comprehensive framework to explain individuals’ achievement-related behaviors (Eccles and Wigfield, 2020). The theory suggests and emphasizes two central constructs: expectancies for success and subjective task values Expectancies for success refer to individuals’ beliefs about their likelihood of succeeding in a given task. These beliefs can be shaped by prior experiences or external feedback (Wigfield and Eccles, 2000). The other component of expectancy-value theory is task value, which refers to the importance individuals attach to a task. The researchers summarize the reasons for engaging in a task under four dimensions. Firstly, people can attend a task because they enjoy it (intrinsic value). Second, the task may help advance the participants’ goals. In other words, it will be useful for the individual. If the task aligns with personal identity, it may also encourage students to engage in the task, (attainment value). Last but not least, it is important to consider, the potential downsides or sacrifices associated with engaging in the task referred to as “cost value” (Eccles et al., 1983; Eccles, 2009; Wigfield and Eccles, 2002; Flake et al., 2015).

Research has shown that students’ expectancies for success (e.g., *academic self-concept*) and task value beliefs take on a leading role on students’ achievement and achievement-related behavior. For example, Wigfield and Eccles (2000) emphasized that task value beliefs, such as intrinsic and utility value, directly contribute to academic achievement by fostering sustained engagement and focus. Similarly, Marsh et al. (2005) found that domain-specific self-concept strongly predicts students’ performance, demonstrating a reciprocal relationship between self-beliefs and achievement. In another study, Guay et al. (2010) showed that students with high academic self-concept in mathematics achieved greater success, as their positive self-beliefs motivated them to exert more effort and persist in challenging tasks.

According to the expectancy-value theory, the interaction of competence and task value can also affect students’ achievement related behaviors (Atkinson, 1957). Nagengast et al. (2011) discussed how the interaction of expectancy and value was at the center of the expectancy-value theory when it was proposed. The original version of the theory underlined that both factors (expectancy and value) should be high for high motivation. However, the interaction effect was dropped, and generally, the effects of expectancy and value beliefs were investigated independently without considering the interaction between them. They investigated the interaction effect with a large sample from 57 countries. According to the findings, the interaction between self-concept and task value was positively and significantly related to achievement-related behaviors for nearly all the participating countries. In line with this, Guo et al. (2015) demonstrated that the interaction between high school students’ math self-concept and their perceived values significantly influenced their achievement-related outcomes such as decisions regarding math course enrollment, academic outcomes, subsequent choices of STEM majors, and university entry pathways. In another study, Guo et al. (2017) investigated expectancy-value beliefs and their interactions from a dimensional comparison perspective. They tested the theory for physics, chemistry, earth science, and biology. The interaction effect was significant for each domain. Building on these established interactions between expectancy and value, the present study applies this framework to examine academic resilience in science among native and immigrant students in Türkiye.

### The present study

This study aims to investigate academic resilience in science among native and immigrant students and to explore the role of motivational factors in the science achievement of non-resilient students. Academic resilience is defined as successful academic performance despite disadvantaged backgrounds and is based on the dimensions of risk and positive additivity (Martin, 2002; Tudor and Spray, 2017). Various factors such as socioeconomic inadequacies, language barriers, family problems and educational inequalities are known to pose risks to students (Waxman et al., 2003; Ungar et al., 2007). In this context, determining the levels of academic resilience of immigrant and native students in science, in Türkiye is crucial for understanding what policies and practices may be necessary for equal opportunities in education (OECD, 2011). Especially considering that immigrant students often face multidimensional risk factors such as cultural adaptation, economic difficulties, and discrimination (Suárez-Orozco et al., 2010; Heath et al., 2008), identifying and supporting the academic resilience levels of these students is important in terms of strengthening the inclusive approach in education.

The other aim of this research is to determine the extent to which motivational beliefs (self-concept and task value) associate to the science performance of non-resilient students from the expectancy-value theory perspective. At this point, it is important to remember that non-resilient students are those who come from low SES families and show low performance in science. As Murdock (1999) underlined, the relation mechanism between motivational beliefs and achievement can be different for low SES students. This underscores the importance of investigating how motivational factors uniquely influence the science achievement of non-resilient immigrant and native students, providing valuable insights into the underlying dynamics of academic resilience in diverse contexts. The interaction of self-concept beliefs and value perceptions will also be considered in the current study. Although the original form of the theory suggests that students’ course success and motivation increase when high self-concept perceptions and high value salience occur together, there are limited studies that investigate this interaction (Nagengast et al., 2011; Guo et al., 2015). To sum up, this study aims to identify motivational strategies to increase the achievement levels of non-resilient students, both migrant and native, particularly in science. To reach these aims, the following research questions will be asked:

1. What is the level of academic resilience in science among native students in Türkiye?
2. What is the level of academic resilience in science among immigrant students in Türkiye?
3. How do motivational factors influence the science achievement of non-resilient immigrant students in Türkiye?
4. How do motivational factors influence the science achievement of non-resilient native students in Türkiye?

## Methods

### Sample

TIMSS 2019 data were used in the current study. TIMSS is an international assessment that measures trends in mathematics and science achievement. In 2019, the Turkish samples consisted of 4,077 eighth-grade students, of whom nearly half (49.3%) were girls and half (50.0%) were boys. 29 students did not report their gender.

In TIMSS, students were asked “were you born in this country?” and “was your mother/father born in this country?.” In this study, students who reported that they or one of their parents were born outside of Türkiye were classified as immigrant students. There were 174 immigrant students in the eighth grade; 142 students either answered they did not know or missed the question. Among immigrant students, 77 were boys; 97 were girls. Since academic resilience is related to students from a low-SES background, participants’ SES was also taken into account. Individuals with a socio-economic background lower than one-third of the group were considered as the low SES group. The frequencies were presented in Table 1.

**TABLE 1:** Distribution of low-SES and non-low-SES participants by gender and immigrant status.

SES Group	Native		Immigrants	
	Girls f (%)	Boys f (%)	Girls f (%)	Boys f (%)
Low SES	564 (30.4%)	674 (35.4%)	32 (33%)	18 (23.4)
Non-low SES	1291 (69.6 %)	1232 (64.6 %)	65 (67%)	59 (76.6)

### Instruments

#### Socio-economic status

Having a personal room or internet connection at home, parents’ educational levels (both mothers’ and fathers’), and the number of books in the household are variables that indicate SES. Parental educational levels were categorized into five options: primary education completed, lower secondary education completed, upper secondary education completed, post-secondary education completed, and university education or higher. The number of books in the home was also categorized into five options: 0–10, 11–25, 26–100, 101–200, and more than 200. TIMSS compiled these questions into a composite variable known as “home educational resources.”

#### Self-concept

Students self-concept was assessed using four items such as *“I usually do well in science*.” It is a four-point Likert scale that spans from “agree a lot” to “disagree a lot.” The reliability coefficients were calculated as 0.67 for immigrant students and 0.89 for native Turkish students in this study.

#### Task value

TIMSS assesses students’ utility value beliefs with eight items, such as “*I think learning science will help me in my daily life*” with a four-point Likert scale. The reliability coefficients were calculated as 0.88 for immigrant and 0.90 for native Turkish students in this study.

#### Academic resilience

Academic resilience relies on two main dimensions: risk and positive additivity (Tudor and Spray, 2017). In this study, risk is represented by SES, while success (positive additivity) is measured through student achievement in science. In the present study, a two-step approach, presented by Teig (2023), is followed to define resilient and non-resilient students. The first step is identifying disadvantaged students. Students in the lowest third of the SES distribution are classified as disadvantaged and form the study sample. In the second step, among the disadvantaged students, academic resilience is determined using different performance thresholds. In other words, students are defined as academically resilient if they belong to the bottom one-third of the SES distribution but achieve scores within the top one-third of the performance distribution.

### Data analysis

TIMSS 2019 data were obtained using the IDB Analyzer (version 4.0), a software tool developed by the International Association for the Evaluation of Educational Achievement (IEA) (IEA, 2023) for analyzing large-scale assessment data. Subsequent analyses were conducted in R (version 4.4.).

To address the first two research questions, academic resilience rates were compared across native and immigrant student groups using Fisher’s exact test, which was preferred over the chi-square test of independence given the small expected frequency in the immigrant-resilient cell (*n* = 1). Effect size was reported as odds ratio with 95% confidence interval.

To address the third and fourth research questions, hierarchical multiple linear regression analyses were conducted separately for native and immigrant student groups, restricted to non-resilient students. Prior to analysis, self-concept and task value were mean-centered. In Stage 1, gender, self-concept, and task value were entered as predictors. In Stage 2, the interaction term (self-concept × task value) was added. Variance inflation factors (VIF) were examined to assess multicollinearity. To further explore the nature of the significant interaction effects, Johnson-Neyman analyses were conducted to identify the regions of task value where the association between self-concept and science achievement was statistically significant (Bauer and Curran, 2005).

All analyses accounted for the complex sampling design of TIMSS through survey-weighted regression using the survey package in R. Results were pooled across five plausible values using Rubin’s rules (Rubin, 1987). Given the small sample size of the immigrant subgroup (*n* = 49), findings for this group should be interpreted as exploratory.

## Results

### RQ_1–2: what is the level of academic resilience in science among native/immigrant students in Türkiye?

To investigate academic resilience in science, students from low SES backgrounds were identified. Among students from low SES families, 50 had an immigrant background. Within the low SES native student group, 210 students demonstrated academic resilience in science. In contrast, only one student with an immigrant background exhibited academic resilience. Since the data are from a complex dataset (TIMSS), sampling weights were used to estimate population characteristics and ensure that findings are representative of the target population. The population estimations and their corresponding standard errors are presented in Table 2.

**TABLE 2:** Population estimates and standard errors of academic resilience in science by student background.

	Native			Immigrants		
Resilience Status	Population estimation	Std. error	%	Population estimation	Std. error	%
Resilient	56.24231	3.98663	17	396.72	396.72	3.1
Non-resilient	274.19697	5.22806	83	12.37375	1.93539	96.9

Fisher’s exact test was conducted to investigate the association between immigrant status and academic resilience in science, given that the expected frequency in the immigrant-resilient cell was below the conventional threshold of 5 required for chi-square approximation. The findings indicated a statistically significant association between immigrant status and academic resilience [*p* = 0.003, OR = 0.11, 95% CI (0.003, 0.63)]. Native students were approximately 9.4 times more likely to be academically resilient than immigrant students. Among native students, 17.0% demonstrated academic resilience in science, compared to only 3.1% of immigrant students. These findings indicate that immigrant status was associated with substantially lower levels of academic resilience in science.

### RQ_3–4: how do motivational beliefs influence the science achievement of non-resilient native/immigrant students in Türkiye?

Multiple linear regression analyses were conducted to investigate how the combination of gender and motivational beliefs, self-concept and task value in science, the science achievement of non-resilient students, examining native and immigrant students separately. Prior to analysis, Variance inflation factors (VIF) confirmed the absence of multicollinearity in all models (VIF range: 1.01–1.17 for native students; 1.09–1.14 for immigrant students). All analyses were conducted using survey-weighted regression to account for the complex sampling design of TIMSS, and results were pooled across five plausible values using Rubin’s rules.

In Stage 1, the main effects model explained 13% of the variance in science achievement for native students and 11% for immigrant students. For both groups, no significant gender differences were observed. Self-concept was the only significant predictor for both native [β = 0.37, 95% CI (0.29, 0.45)] and immigrant students [β = 0.31, 95% CI (0.04, 0.58)]. Task value was not significantly associated with science achievement in either group. The regression coefficients are presented in Table 3.

**TABLE 3:** Stage 1: main effects of self-concept, task value, and gender on science achievement.

Variable	Native students				Immigrant students			
	*b*	SE	β	95% CI	*b*	SE	β	95% CI
Intercept	452.85	9.36	–	[434.38, 471.32]	396.88	46.90	–	[304.53, 489.22]
Gender	−5.59	5.22	−0.04	[−15.84, 4.65]	−9.84	31.86	−0.05	[−72.48, 52.80]
Self-concept	15.24	1.65	0.37*	[11.95, 18.54]	18.71	8.18	0.31*	[2.50, 34.91]
Task value	−2.25	1.60	−0.06	[−5.50, 1.01]	−6.02	10.36	−0.12	[−26.57, 14.52]
R^2^	0.13				0.11			

Predictors are mean-centered. b, unstandardized coefficient; SE, standard error; β, standardized coefficient; CI, confidence interval.

*p < 0.05.

In Stage 2, the interaction term between self-concept and task value was added to the model. The model explained 14% of the variance for native students and 21% for immigrant students, with the interaction term accounting for an additional 1 and 10% of variance, respectively. Gender remained non-significant for both groups. Self-concept continued to be a significant positive predictor for both native [β = 0.36, 95% CI (0.28, 0.43)] and immigrant students [β = 0.36, 95% CI (0.08, 0.64)]. Task value remained non-significant for both groups.

Regarding the interaction effect, the pattern diverged markedly between the two groups. For native students, the interaction between self-concept and task value was positive and statistically significant [β = 0.09, 95% CI (0.02, 0.15)], indicating that the positive association between self-concept and science achievement was stronger when task value was also higher. For immigrant students, however, the interaction was negative and statistically significant [β = −0.35, 95% CI (−0.63, −0.08)], suggesting that the positive association between self-concept and science achievement weakened—and eventually reversed—as task value increased. The regression coefficients are presented in Table 4.

**TABLE 4:** Stage 2: interaction model of self-concept × task value on science achievement.

Variable	Native students				Immigrant students			
	*b*	SE	β	95% CI	*b*	SE	β	95% CI
Intercept	450.12	9.17	–	[432.06, 468.19]	413.65	43.53	–	[327.90, 499.40]
Gender	−5.24	5.13	−0.03	[−15.31, 4.82]	−19.00	29.64	−0.10	[−77.31, 39.32]
Self-concept	14.71	1.61	0.36*	[11.48, 17.94]	21.72	6.64	0.36*	[8.42, 35.01]
Task value	−1.44	1.52	−0.04	[−4.51, 1.63]	−10.39	9.21	−0.20	[−28.72, 7.95]
Self-concept × Task value	1.65	0.67	0.09*	[0.32, 2.98]	−12.72	5.22	−0.35*	[−23.00, −2.44]
R^2^	0.14				0.21			
ΔR^2^	0.008				0.10			

Predictors are mean-centered. SC × TV = self-concept × task value interaction term. B, unstandardized coefficient; SE, standard error; β, standardized coefficient; CI, confidence interval.

*p < 0.05.

**FIGURE 1:**
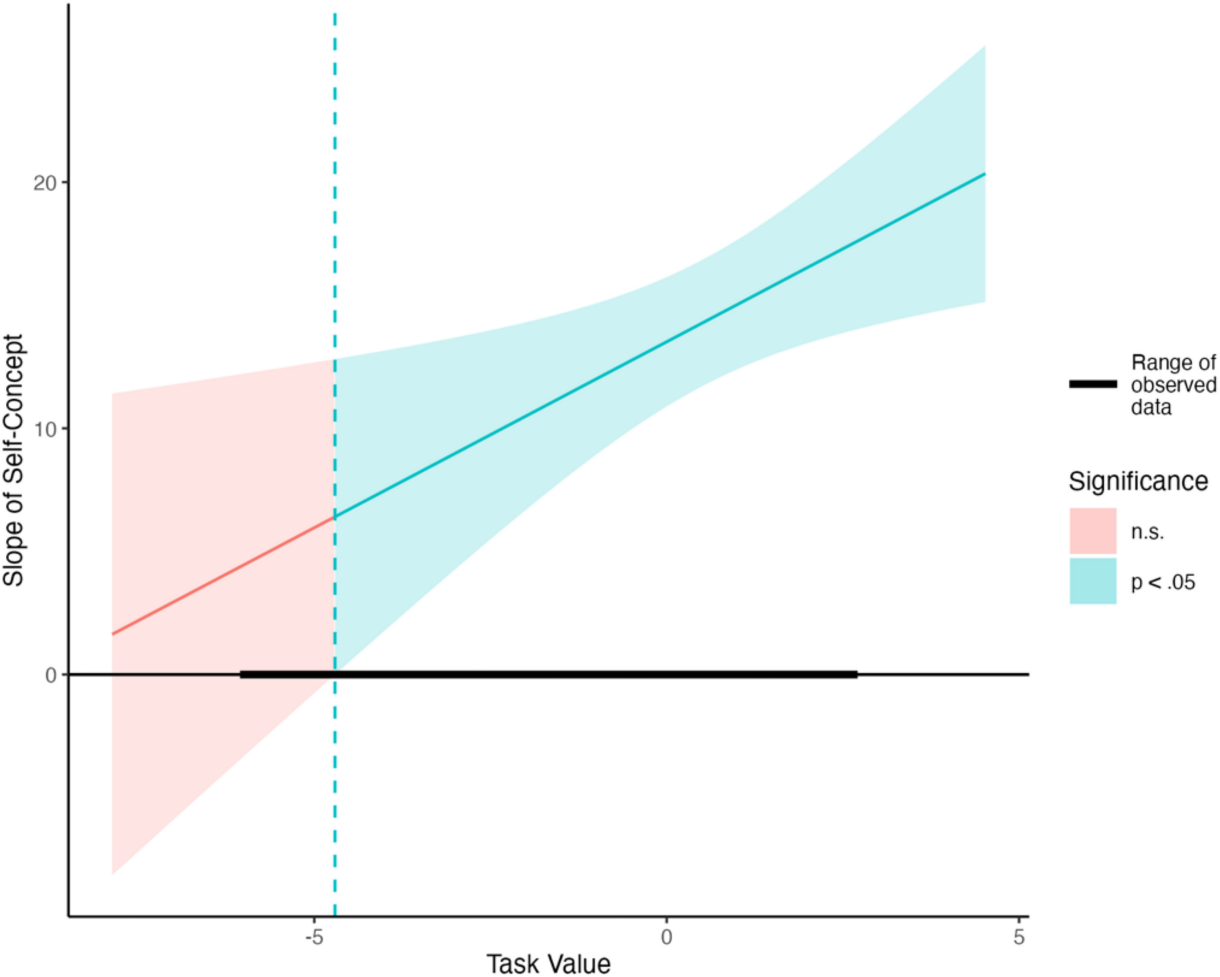
Johnson-Neyman plot for native non-resilient students.

Johnson-Neyman analyses indicated that among native students, the association between self-concept and science achievement was significant when task value was above −4.71, which encompasses nearly the entire range of observed task value values (−6.00, 2.65). The Johnson-Neyman plot for native students is presented in Figure 1.

**FIGURE 2:**
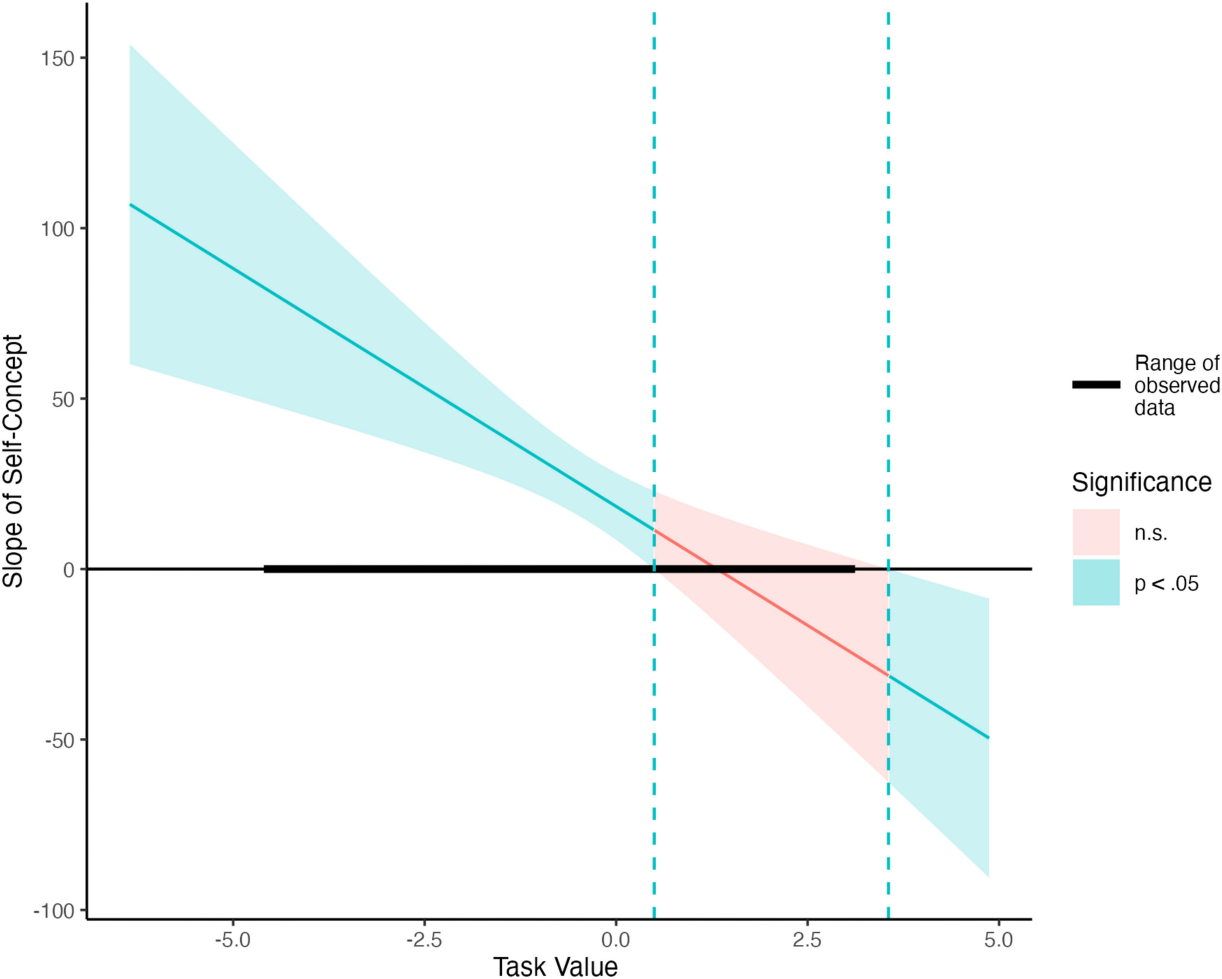
Johnson-Neyman plot for immigrant non-resilient students.

For immigrant students, the analyses indicated that among immigrant students, the association between self-concept and science achievement was positive and significant when task value was below 0.50, non-significant between 0.50 and 3.57, and negative and significant above 3.57. The Johnson-Neyman plot for immigrant students is presented in Figure 2.

## Discussion

This study aimed to explore academic resilience in science by examining both native and immigrant students and investigating how motivational factors, from the expectancy-value perspective, are associated with the science achievement of non-resilient students. Using TIMSS 2019 data from Türkiye, the findings reveal a substantial gap in academic resilience between the two groups, as well as divergent motivational patterns.

### Academic resilience among native and immigrant students

According to the findings, there is a very low proportion of academically resilient students among the immigrant group, compared to native students from similarly disadvantaged (low SES) backgrounds. This aligns with prior literature indicating that that immigrant students frequently encounter language barriers, economic hardships, cultural adaptation challenges, and discrimination, all of which are associated with lower academic trajectories (Suárez-Orozco et al., 2010). Consistent with Gabrielli et al. (2022), who reported that immigrant students across European countries systematically demonstrate lower academic resilience than native peers, the present findings extend this pattern to the Turkish context, where immigrant students face additional linguistic and cultural barriers. While the academic resilience concept underscores that some students still manage to succeed against these odds (Martin, 2002; Tudor and Spray, 2017), the findings suggest that these challenging contexts may inhibit the protective processes that are associated with resilience in Turkish schools. One possible explanation is that immigrant students may have a lower baseline of educational resources and social support, exacerbated by cultural and linguistic mismatches (Heath et al., 2008). Additionally, resilience develops through the interplay between individuals and their environments (Ungar et al., 2007), and structural barriers such as insufficient language support or lack of culturally responsive instruction may weaken this process for immigrant students. Supporting this idea, Çelik and İçduygu (2019) found that refugee students in Türkiye have been found to face systematic language barriers and institutional challenges in public schools. Consistent with OECD (2011) reports, the significant gap in resilience highlights the need for systemic educational policies aimed at mitigating the adverse effects of socioeconomic and cultural challenges for immigrant students.

### Relation between motivational beliefs and science achievement for non-resilient students

The other aim of the study was to investigate how motivational factors related to non-resilient middle school students’ science achievement, using the expectancy-value theory as a framework, which emphasizes the role of students’ beliefs about their abilities (self-concept) and the value they assign (task value) to science learning (Wigfield and Eccles, 2000). The initial regression models suggest that self-concept was positively and significantly associated with science achievement for both native and immigrant non-resilient students. These findings are consistent with the expectancy-value framework, where positive self-beliefs often encourage greater effort and persistence, thus improving academic outcomes (Marsh et al., 2005; Wigfield and Eccles, 2000). For students already struggling academically, perceiving themselves as capable in science may serve as a critical motivational buffer that is associated with high engagement with the subject despite low SES or other barriers (Guay et al., 2010). Interestingly, task value did not show a significant positive effect in the first set of models for either group. This result diverges somewhat from classic expectancy-value research, which generally posits a robust link between task value and achievement (Wigfield and Eccles, 2000; Eccles, 2009). One explanation could be that students with limited academic success, particularly those facing consistent structural or socio-cultural barriers, may find it challenging to translate the subjective importance or utility of science into performance gains. Perceived cost factors such as time, effort, and emotional burden may also attenuate the positive association of task value for these students; however, since cost was not directly measured in TIMSS, future studies could examine this possibility using validated cost-dimension scales.

Since expectancy-value theory suggests that the interaction of self-beliefs and task value can also affect students’ achievement-related behaviors (Atkinson, 1957), in the following phase of this study, the interaction term of self-concept and task value was also added to the model. When the interaction term was added to the model, the pattern diverged markedly between the two groups. For native students from low SES backgrounds, the interaction of self-concept and task value was positively related to science achievement. This suggests that having both a strong sense of one’s scientific capabilities (expectancy) and perceiving science as valuable or useful (value) can be associated with higher achievement, mirroring the core proposition in the original expectancy-value framework (Atkinson, 1957; Nagengast et al., 2011).

However, when the interaction term was added, the association between self-concept and achievement was no longer significant, and task value was negatively associated with achievement. This pattern implies a compensatory or interdependent dynamic: high self-concept alone or high task value alone may not suffice for these disadvantaged native students, but in combination, they are associated with higher achievement. By contrast, for immigrant non-resilient students, self-concept and task value each had significant positive main effects, but their interaction was negative. This was an unexpected finding since Nagengast et al. (2011) reported a positive expectancy × value interaction for Türkiye and nearly all 57 countries in their cross-national study. One explanation may be that for these students, holding both high self-concept and high task value simultaneously might create tensions or incongruities in practice. For instance, an immigrant student might strongly believe in their ability (high self-concept) and see science as worthwhile (high task value), but still lack adequate linguistic or institutional support to capitalize on these motivational beliefs. When structural or cultural barriers limit students’ ability to convert these beliefs into achievement gains, the co-occurrence of high expectancy and high value may not translate into higher performance. Perceived cost factors such as time, effort, and emotional burden may also play a role; however, since cost was not directly measured in TIMSS, future studies could examine this possibility using validated cost-dimension scales. While the classic expectancy-value framework predicts a positive interplay between high expectancy and high value (Nagengast et al., 2011; Guo et al., 2015), the present findings suggest that linguistic, cultural, and systemic factors may alter how these motivational beliefs function together for immigrant students. Actually, this finding is consisted with Kaya et al. (2024), who found that self-theories such as resilience operate differently for immigrant students across national contexts

There are some limitations to this study. Firstly, this study uses descriptive and correlational designs, and it does not claim a cause-and-effect relationship. The term “effect” means statistical effect, not a causal effect. Furthermore, the cross-sectional design precludes definitive causal inferences; longitudinal studies could more effectively clarify how resilience and motivational beliefs evolve over time. Therefore, in a further study, these concepts can be investigated using a longitudinal design. Second, the number of immigrant students who were both low SES and academically resilient was very small, which limits the statistical power and generalizability of subgroup analyses. The relatively small sample size in this subgroup (*n* = 49) is particularly limiting for regression models including interaction terms, which typically require substantially larger samples to achieve stable and reliable estimates. In particular, interaction effects in small samples are sensitive to model specification and may be unstable. Although the study provides useful descriptive insights into educational resilience among disadvantaged students, future research should replicate these analyses using larger and more targeted immigrant samples. Additionally, the number of immigrant students classified as academically resilient was extremely small (*n* = 1), which limits the reliability of group-level comparisons and warrants particular caution in interpreting the resilience gap findings. Next, this study is based on TIMSS 2019 data; although TIMSS 2023 data became publicly available in December 2024, the present study was substantially completed by that time, and replication with the more recent dataset is encouraged. Finally, future research could benefit from mixed-methods approaches, such as interviews or classroom observations, to provide deeper insight into the mechanisms underlying motivational processes among immigrant students, particularly in relation to variations in self-concept and task value.

## Conclusion

This study examined academic resilience in science among native and immigrant students in Türkiye and investigated how motivational beliefs are associated with science achievement among non-resilient students. The findings reveal a substantial gap between the two groups. According to the results, native students were approximately 9.4 times more likely to demonstrate resilience than their immigrant peers. Self-concept was positively associated with science achievement in both groups, while the interaction of self-concept and task value showed opposite patterns: positive for native students and negative for immigrant students. This suggests that the same motivational beliefs may function differently depending on the structural and cultural circumstances students face. Expanding language support and culturally responsive instruction for low-SES immigrant students may help address this gap. Future studies should adopt longitudinal designs and include direct measures of perceived cost and acculturation to better understand these dynamics.

## Data Availability

Publicly available datasets were analyzed in this study. This data can be found at: https://timssandpirls.bc.edu/timss2019/international-database/.
